# Performance of Traditional Cardiovascular Risk Scores and Objective Optimization in Cancer Survivors

**DOI:** 10.3390/curroncol33040230

**Published:** 2026-04-19

**Authors:** Harsh A. Patel, Saifullah Syed, Pranathi Tella, Harshith Thyagaturu, Brijesh Patel

**Affiliations:** 1 Department of Internal Medicine, Geetanjali Medical College and Hospital, Udaipur 313001, India; 2 Department of Internal Medicine, Indiana University School of Medicine, Indianapolis, IN 46202, USA; 3 Department of Internal Medicine, P.S.I. Medical College and Hospital, Chinoutpalli 521286, India; 4 Department of Cardiovascular and Thoracic Surgery, Heart and Vascular Institute, West Virginia University, Morgantown, WV 26506, USA; 5 Division of Cardiovascular Medicine, Krannert Cardiovascular Research Center, Indiana University, Indianapolis, IN 46202, USA

**Keywords:** cardiovascular risk scores, cancer survivors, National Health and Nutrition Examination Survey (NHANES), cardiovascular mortality, Youden Index

## Abstract

**Introduction:** Cardiovascular disease (CVD) is a leading cause of non-cancer death among cancer survivors, attributable to cardiotoxic therapies and cardiovascular risk factors. General population risk prediction tools, including ASCVD (Atherosclerotic cardiovascular disease), Framingham’s Score, and PREVENT (Predicting Risk of Cardiovascular Disease EVENTS), lack cancer-specific variables. We evaluated whether these models, even after statistical optimization, could predict cardiovascular mortality in cancer survivors. **Methods:** Using the National Health and Nutrition Examination Survey (NHANES) 2001–2018, linked with National Death Index (NDI) mortality data, we conducted a retrospective analysis of 634 and 429 cancer survivors, respectively, across model-specific cohorts free of baseline cardiovascular disease. Discrimination was assessed for ASCVD, Framingham Score, and PREVENT using standardized thresholds of 7.5% and 20%, as well as Youden-optimized cutoffs. Area under the curve (AUC) comparisons were performed using the DeLong non-parametric method. **Results:** Standard thresholds showed suboptimal discrimination across all models (AUCs: ASCVD 0.56, Framingham 0.53, PREVENT 0.64). In contrast, Youden-optimized AUCs (ASCVD: 0.68; PREVENT: 0.71; all *p* < 0.001, DeLong test). Optimization increased the “low-risk” group’s mortality rate from 2.8% to 4.1% (RR = 1.47), suggesting improved statistical fit came at the cost of overestimating the risk. Optimized thresholds outperformed conventional cutoffs, underscoring the necessity for recalibrated, cohort-specific risk stratification in cancer survivors. **Conclusions:** Standard risk scores have inadequate discrimination for cardiovascular mortality prediction in cancer survivors. Threshold recalibration improves statistical metrics but does not resolve the structural failure of these models to account for cardiotoxic exposure. Development of cardio-oncology-specific risk models incorporating oncologic exposures is therefore warranted.

## Simple Summary

Cancer survivors are living longer but face a higher risk of heart disease compared to the general population, partly due to cancer treatments such as chemotherapy. Doctors rely on standard heart risk calculators to predict problems and determine who may benefit from preventive therapies like statins. However, it remains unclear if these tools accurately predict risk for people with a history of cancer. Using national health data, we evaluated three ASCVD risk scores (PCE, Framingham, and PREVENT) and found they all performed poorly in cancer survivors. When we recalibrated the tools to perform better statistically, the improvement came with a serious drawback: more high-risk survivors were incorrectly labeled as low risk. Modifying existing heart risk calculators is insufficient. We need new risk models designed specifically for cancer survivors that incorporate their unique treatment history and cancer-related variables. This will help guide better prevention strategies and improve long-term cardiovascular care.

## 1. Introduction

Advances in oncology have led to a growing population of more than 18 million cancer survivors in the United States, many of whom are living long enough to experience chronic cardiovascular complications [1,2]. In this setting, cardiovascular disease (CVD) has emerged as the leading cause of noncancer death among cancer survivors [3]. The excess risk is driven by the intersection of traditional cardiovascular risk factors, systemic inflammation, accelerated atherosclerotic processes, and the cardiotoxic effects of life-saving cancer therapies, including anthracyclines, trastuzumab, and radiation [4,5]. Accurate, long-term cardiovascular risk stratification is therefore essential to guide timely preventive strategies and improvement of overall survival in this population.

Cardiovascular risk assessment has evolved from older models such as the Framingham Risk Score (FRS) [6], estimating 10-year cardiovascular event risk using age, sex, cholesterol, blood pressure, smoking, and diabetes to the 2013 ACC/AHA Pooled Cohort Equations (Atherosclerotic Cardiovascular Disease [ASCVD] risk calculator) [7], which incorporated race to guide statin initiation at a 7.5% threshold, and most recently to the American Heart Association’s Predicting Risk of Cardiovascular Disease Events (PREVENT) equations (2023) [8], which further integrate estimated glomerular filtration rate (eGFR) and social determinants of health to project 10- and 30-year risk in asymptomatic adults. While these tools perform well in their origin cohorts, these models are structurally limited because they do not integrate cancer-specific data such as treatment-related cardiotoxicity, cumulative therapeutic dose or survivorship-related risk trajectories. As a result, they may not reflect the true cardiovascular risk faced by cancer survivors and may lead to misclassifying high-risk patients as low-risk and delaying appropriate cardioprotective care.

While prior studies have documented the poor-to-moderate discrimination of conventional cardiovascular risk scores in cancer survivors, most investigations focused exclusively on discrimination metrics [9,10]. To our knowledge, this is the first study to evaluate the impact of statistical recalibration of conventional risk scores in nationally representative U.S. cancer survivor cohorts. Limited data exist on whether statistical recalibration of these tools can translate to a real-world benefit in patient care and classification. We addressed this specific gap by evaluating not only whether ASCVD, PREVENT, and FRS could discriminate cardiovascular mortality in cancer survivors at standard thresholds, but also whether Youden Index optimization (J statistic) [11], which identifies the cutpoint maximizing the sum of sensitivity and specificity, can meaningfully improve clinical risk classification in this population.

We hypothesized that these general population tools would demonstrate limited discriminative performance for cardiovascular mortality in cancer survivors. We also examined whether mathematical recalibration using the Youden Index could improve model discrimination for clinical use or show whether underlying structure limits their utility in cardio-oncology practice.

## 2. Methods

### 2.1. Data Sources and Sampling

This retrospective study utilized de-identified data from the National Health and Nutrition Examination Survey (NHANES), a series of 2-year cross-sectional surveys conducted by the National Center for Health Statistics (NCHS) of the U.S. Centers for Disease Control and Prevention [12]. We included nine consecutive NHANES cycles spanning 2001 through 2018. NHANES uses a complex, stratified, multistage probability sampling design to obtain a sample representative of the non-institutionalized U.S. civilian population. The protocols, methodology, and data are publicly available [13]. All cycles were approved by the NCHS Research Ethics Review Board, and every participant provided written informed consent.

We utilized the NHANES-linked mortality file by linking participant data to the National Death Index (NDI) with follow-up available through 31 December 2019, for cardiovascular mortality [14]. All proportions presented utilize appropriate NHANES sample weights, as recommended by NHANES reporting guidelines.

### 2.2. Data Collection

Data were collected via trained interviewers who administered standardized questionnaires in participants’ homes, followed by a comprehensive physical and laboratory assessment conducted in mobile examination centers (MEC).

Baseline information on age, sex, race/ethnicity, and self-reported history of smoking status (Do you now smoke cigarettes?), hypertension (Ever told you had high blood pressure?), heart failure (Ever told had congestive heart failure?), stroke (Ever told you had a stroke?), coronary artery disease (Ever told you had coronary heart disease?), and cancer (Ever told you had cancer or malignancy?) were obtained by standard questionnaire. Medication use, including antihypertensive medications (Now taking prescribed medicine for high blood pressure) and statins (a derived variable for statin use based on prescription medication data), was validated via pill bottle review.

Objective measurements included blood pressure, which was measured by trained personnel using an Omron HEM–907XL digital upper-arm electronic device following a five-minute seated rest. The final blood pressure value for analysis represents the average of the second and third of three consecutive measurements taken 60 s apart. Body Mass Index (BMI) was calculated from collected height and weight measurements.

Blood laboratory samples were collected during mobile examinations, with analysis of hemoglobin A1c performed on all samples. Analysis of triglyceride, LDL, HDL, and total cholesterol was performed only on participants who completed a fast of 8 to 24 h, and participants with non-fasting lipid values were excluded from lipid-specific analyses to maintain the validity of the risk score calculations.

### 2.3. Study Population

From an initial cohort of 91,351 NHANES participants across the 2001–2018 cycles. To align with the primary prevention framework, participants with baseline cardiovascular disease (coronary artery disease, heart failure, myocardial infarction, or stroke) were excluded, which resulted in a general primary prevention cohort of 19,368 participants. Subsequent exclusions for missing key variables (cancer status, diabetes, and hypertension) necessary for risk factor calculation yielded a final eligible general cohort of 3919 individuals. Within this final cohort, a dedicated cancer survivor sub-cohort of 634 individuals (weighted N = 2,937,598) was identified for the primary analysis.

Three risk scores were calculated: ASCVD (Pooled Cohort Equation 2013), the PREVENT score (eGFR calculated using the 2021 CKD-EPI eGFR equation), and the Framingham Risk Score [15]. The Framingham 10-year cardiovascular risk score was calculated using the general cardiovascular disease prediction model developed by D’Agostino et al. [15]. Variables required for the model (age, sex, systolic blood pressure, antihypertensive treatment status, smoking status, diabetes, HDL cholesterol, and total cholesterol) were mapped to corresponding NHANES variables.

Because NHANES does not provide a pre-calculated Framingham score, the risk equation was implemented using the Stata “Framingham” command, which reproduces the published Framingham coefficients. The predicted 10-year risk was expressed as a percentage and analyzed as both a continuous variable and categorical thresholds.

To ensure model-specific validity, analysis was performed in separate sub-cohorts based on each score’s unique age and exclusion criteria. The ASCVD and Framingham models were evaluated in 634 survivors (weighted N = 2,937,598), while the PREVENT score was analyzed in 429 survivors (weighted N = 2,113,856) (Table 1). Participants with missing data for any variable required to calculate a given risk score were excluded from that model-specific analysis, as complete covariate information is necessary for valid risk score estimation.

### 2.4. Outcomes

Cardiovascular mortality served as the binary discrimination endpoint against which the predictive performance of each risk score was evaluated, which was determined by linking participant data to the NHANES-linked mortality file (National Death Index, NDI). Deaths were categorized as cardiovascular and cerebrovascular based on the underlying cause of death being coded according to the International Classification of Diseases, Tenth Revision (ICD-10) codes.

The time to event for each participant was calculated as the number of months from the date of the NHANES examination until the date of cardiovascular mortality or the end of the follow-up period (31 December 2019), whichever occurred first.

### 2.5. Statistical Analysis

All analyses accounted for the complex NHANES survey design using the svy framework in Stata, incorporating examination sample weights (WTINT2YR), primary sampling units (SDMVPSU), and strata (SDMVSTRA) to ensure nationally representative estimates.

Model Performance: Risk discrimination was quantified using Receiver Operating Characteristic (ROC) analysis and the Area Under the Curve (AUC). AUCs were compared using the DeLong non-parametric method (roccomp, Stata v19.0)

Risk ratios were estimated using survey-weighted Poisson regression with robust variance. Model calibration for the association between continuous risk scores was assessed using the Hosmer–Lemeshow goodness-of-fit test. To assess the clinical utility and incremental value of the risk scores, we calculated the Net Reclassification Index (NRI) and the Somers’ D statistics. We evaluated score performance at three distinct 10-year risk thresholds: the conventional 7.5% threshold, a high-risk 20% cutoff, and a Youden-optimized threshold calculated using the STATA “cutpt” command for maximizing the sum of specificity and sensitivity.

Statistical significance was set at a two-sided *p* value < 0.05. All statistical analyses were performed using Stata version 19.0 BE-Standard Edition (StataCorp, College Station, TX, USA).

## 3. Result

Among 634 cancer survivors in the ASCVD and FRS cohorts (weighted N = 2,937,598), 50 cardiovascular deaths were observed (weighted event rate 6.76%). In the PREVENT cohort (n = 429; weighted N = 2,113,856), 26 cardiovascular deaths occurred (weighted event rate 4.33%). Overall, traditional cardiovascular risk scores demonstrated poor-to-moderate discrimination for cardiovascular mortality. At the standard clinical threshold (7.5%), the ASCVD yielded an AUC of 0.56 (95% CI 0.53–0.59), while the Framingham Risk Score performed near chance (AUC 0.53, 95% CI 0.49–0.57) (Table 2). Although the PREVENT score demonstrated the highest baseline performance among the three (AUC 0.64, 95% CI 0.57–0.71), it remained suboptimal for definitive clinical risk stratification.

**Figure 1:**
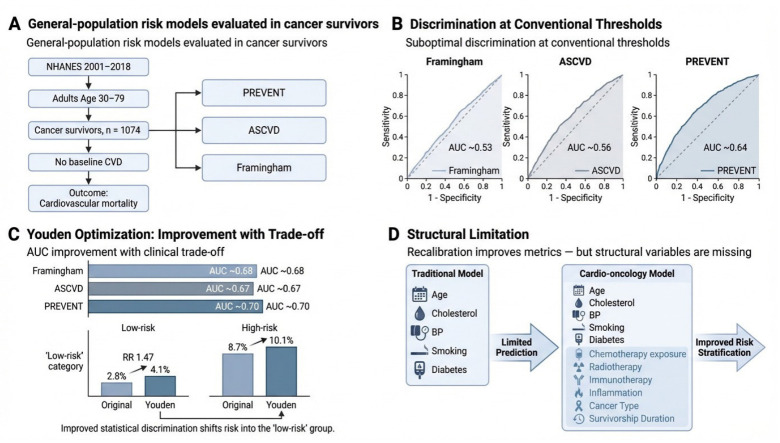
Using NHANES 2001–2018 data linked to the National Death Index, we assessed the performance of general population risk tools (PREVENT, ASCVD, and Framingham) in predicting cardiovascular mortality among cancer survivors. At standard thresholds, all models showed limited accuracy. Recalibration using the Youden index improved statistical performance but introduced a clinically relevant issue, with higher mortality observed within the “low risk” category. This finding suggests that the limitation lies in model design, likely due to the absence of oncology-related variables, rather than threshold selection alone. These results support the need for risk models specifically developed for cardio-oncology populations.

Recalibrating the models using the Youden Index yielded statistical improvements in discrimination across all models. With the optimal ASCVD cutoff shifted to 19.46%, the AUC to 0.68. Similarly, Framingham’s cutoff was 30.25% (AUC: 0.66), and PREVENT’s was 11.92% (AUC: 0.70). (Figure 1B,C). Overall differences across thresholds were statistically significant (ASCVD: χ^2^ = 20.99, *p* < 0.001; FRS: χ^2^ = 23.65, *p* < 0.001; PREVENT: χ^2^ = 16.18, *p* = 0.0003, DeLong method).

However, this statistical optimization has shifted in risk categorization. In the PREVENT model, the Youden-derived cutoff improved the separation of risk groups (Somers’ D 0.41 vs. 0.29). It simultaneously increased the mortality rate within the “low-risk” tier. Specifically, cardiovascular mortality among survivors labeled “low risk” rose from 2.8% at the original cutoff to 4.1% at the Youden-optimized cutoff (RR 1.47), representing a 47% increase in missed fatal events. While it increased, mortality in the “high risk” group rose to 10.1% from 8.7%.

Continuous 10-year cardiovascular risk scores were significantly associated with cardiovascular mortality across all three models: ASCVD RR 1.03 (95% CI 1.02–1.04); FRS RR 1.03 (95% CI 1.02–1.05); and PREVENT RR 1.15 (95% CI 1.09–1.21), all *p* < 0.001. Calibration assessment of the continuous PREVENT score demonstrated poor absolute risk estimation in this population (Hosmer–Lemeshow F (9408) = 2054.30; *p* < 0.001), consistent with systematic miscalibration when a general population tool is applied to cancer survivors.

At the Youden-optimized categorical threshold, high or intermediate risk classification was associated with significantly higher risk of cardiovascular death across all three models: ASCVD RR 7.27 (95% CI 2.94–18.02); FRS RR 5.05 (95% CI 2.61–9.75); and PREVENT RR 7.99 (95% CI 2.89–22.09), all *p* < 0.001. The wide confidence intervals reflect the modest event counts in each cohort and should be interpreted accordingly.

Despite these metrics, net reclassification analysis revealed that the “gain” in model performance was driven almost entirely by the better identification of non-events (NRI ≈ +0.27). In contrast, the identification of actual fatal events worsened (NRI ≈ −0.15). The modest total NRI (+0.11) underscores that threshold optimization largely reclassifies survivors who would survive anyway, rather than capturing the vulnerable patients that traditional models overlook.

## 4. Discussion

This study asked whether three widely used cardiovascular risk prediction models, ASCVD, Framingham Risk Score, and PREVENT, could stratify cardiovascular mortality risk in cancer survivors, and whether statistical optimization could improve the performance when the standard threshold fell short. The answer to both questions is instructive. First, all three models showed poor-to-moderate discrimination at standard clinical thresholds, with AUC ranging from 0.535 to 0.646. Second, Youden recalibration improved AUC estimates across all models yet compromised clinical safety by increasing cardiovascular mortality in the low-risk stratum from 2.8% to 4.1% (47% increase), and Third, the net reclassification analysis confirmed that the statistical gain from optimization was almost entirely due to improved identification of survivors who would not die, while the detection of fatal events declined. Taken together, these findings confirm that threshold manipulation cannot overcome the fundamental absence of oncologic variables in these models and provide data-driven support for the development and validation of dedicated cardio-oncology risk prediction models. To our knowledge, this is the first demonstration in a nationally representative US cancer survivor’s cohort that Youden optimization- the most commonly applied method for threshold recalibration- does not improve clinical risk classification and may actively worsen it [16] (Figure 1).

The clinical implications of threshold optimization highlight a fundamental paradox. The Youden Index is a well-established method for maximizing combined sensitivity and specificity at a single cutpoint [11]. Although the Youden-derived cutoff values yielded better discrimination, they were obtained at the cost of patient safety through systematic downward risk reclassification. Specifically, when using the optimized PREVENT threshold (11.92% vs. 7.5%), the cardiovascular mortality rate in the “low risk” stratum increased from 2.8% to 4.1%, a relative increase of 47%.

On the other hand, the corresponding increase in mortality in the “high risk” stratum was relatively small (8.7% to 10.1%), confirming that the optimization algorithm primarily shifts event survivors into the “low risk” category rather than improving the detection of high-risk patients. The net reclassification improvement further supports this interpretation: although classification of non-events improved (NRI ≈ +0.27), it worsened the detection of fatal events (NRI ≈ −0.15). These findings suggest that threshold optimization, although mathematically attractive, does not solve the underlying problem, the absence of structural variables necessary to capture cardio-oncology-specific risk. Reliance on general population cutoffs risks systematic misclassification, either underestimating risk and missing opportunities for primary prevention (Statin/ACE-inhibitors initiation) or overestimating risk in these high-risk groups, with unnecessary treatment and anxiety.

Traditional cardiovascular risk scores rely on well-established atherosclerotic risk factors: age, sex, lipid profiles, blood pressure, and glycemic status. While these factors remain prognostically relevant in cancer survivors, they are unable to account for the “accelerated vascular aging” and non-traditional pathophysiologic mechanisms of disease unique to oncology. For example, Anthracycline-induced cardiomyopathy, common in breast cancer survivors, operates through direct topoisomerase II-mediated myocardial injury and mitochondrial oxidative stress, independent of atherosclerotic burden [17]. Trastuzumab-related cardiotoxicity arises via HER2-pathway disruption in cardiomyocytes, while thoracic radiation accelerates coronary artery disease through endothelial inflammation, intimal hyperplasia, and pericardial fibrosis [18]. Immune checkpoint inhibitors, increasingly deployed across cancer types, have been implicated in immune-mediated myocarditis with mortality rates exceeding 50% in severe cases [19]. These mechanisms are invisible to current risk tools, which assume a linear progression of risk that cancer therapy fundamentally disrupts.

Our findings are consistent with a growing body of literature challenging the inadequacy of general-population cardiovascular risk prediction models in oncology. Alvi et al. (2022) reported that in patients with head and neck cancer who receive radiation therapy, standard risk tools (PCE, FRS, USPSTF) underestimated observed cardiovascular event rates by nearly 70%, with patients classified as “low risk” experiencing event rates comparable to those classified as high risk in the general population [20], a finding remarkably similar to the 47% increase in low-risk stratum mortality observed in our study after Youden optimization.

Similarly, Stabellini et al. (2024) demonstrated that the pooled cohort equations had limited discrimination (AUC ~0.61) for atherosclerotic events in prostate cancer survivors [21]. In contrast, a cancer-specific model incorporating androgen deprivation therapy exposure (androgen deprivation therapy (ADT) duration, cumulative ADT exposure, baseline PSA, and Gleason score alongside traditional CVD risk factors) achieved substantially better performance (AUC ~0.71). This 0.10 AUC improvement, similar to the improvement we observed with Youden optimization, suggests that the inclusion of cancer-specific variables provides more clinically significant discrimination than mathematical optimization of existing risk prediction tools. The UK Biobank analysis by McCracken et al. (2024) further supports this perspective, demonstrating suboptimal discrimination across QRISK3, the Framingham Risk score, and SCORE2 in cancer survivors (AUCs of 0.61–0.68), with systematic underestimation of observed events [22]. Crucially, when cancer history variables were deliberately included in QRISK3, discrimination improved, and bias decreased, suggesting that the answer lies in model reformulation rather than threshold calibration. This is further supported by Strongman et al. (2023), who demonstrated that adding cancer history variables in QRISK3 resulted in a net reclassification improvement of 3.9%, with the largest gains in those with recent cancer diagnoses or cardiotoxic therapy [23]. This confirms our hypothesis that oncologic exposures, not cancer diagnoses per se, are the key missing variables. Our study adds to this literature by demonstrating, for the first time in a nationally representative U.S. sample, that the very process of statistical optimization intended to correct for model underperformance actively worsens the clinical classification of high-risk cancer survivors. This finding has direct implications for guideline development and clinical implementation.

### Future Directions and Research Priorities

Cardio-oncology guidelines increasingly emphasize individualized risk assessment and present findings that highlight the interconnected priorities for the field [24,25]. First, the development and validation of cancer-specific cardiovascular risk prediction models in large, diverse populations with extensive cancer treatment information and long-term cardiovascular outcomes. Collaborative projects involving multiple institutions, integrating cancer registries (SEER, NCDB) with cardiovascular outcomes databases (Medicare, EHRs, cardiovascular registries). Machine learning algorithms, such as random forests, gradient boosting, and deep learning, may uncover non-linear relationships between conventional risk factors and cancer-specific variables that are not apparent using linear models [26].

Second, integrating novel biomarkers may enhance model discrimination and enable early intervention. Circulating cardiac troponin and natriuretic peptides (BNP/NT-proBNP) are already incorporated in cardio-oncology surveillance guidelines and have demonstrated incremental prognostic value in treatment-naive cancer populations [27]. Emerging biomarkers, including growth differentiation factor-15 (GDF-15), soluble ST2, galectin-3, and circulating microRNAs, may further refine the detection of subclinical cardiotoxicity, particularly in patients with preserved ejection fraction [28].

Third, imaging-based risk stratification represents an underutilized but high-yield adjunct to clinical risk prediction [29]. Coronary artery calcium scoring provides direct quantification of atherosclerotic burden and has demonstrated independent prognostic value in cancer survivors treated with thoracic radiation [30]. Echocardiographic global longitudinal strain and cardiac magnetic resonance T1/T2 mapping enable detection of subclinical myocardial injury antecedent to a decline in ejection fraction [31,32].

Fourth, adding key variables could enhance cardiovascular risk prediction of future cardio-oncology tools, including: anthracycline cumulative dose, radiation field and dose, immunotherapy exposure, particularly immune checkpoint inhibitors, and time elapsed since treatment [33,34,35,36]. These additions provide concrete, actionable guidance for future model development efforts.

## 5. Limitations

This study had several methodological limitations that warrant acknowledgement. The NHANES relies on self-reported cancer diagnoses and lacks information about detailed cancer type, stage, specific treatment regimens (chemotherapy type, radiation dose), and time since diagnosis [13]. But NHANES remains the only nationally representative dataset with linked mortality data that permits this analysis at the population scale. The exclusion of participants with baseline cardiovascular disease, while necessary to align with the primary preventive framework of the evaluated risk scores, introduces survivorship bias: as cancer survivors who had already experienced cardiovascular complications were excluded, potentially underestimating the true magnitude of risk misclassification in a real-world clinical population where such comorbidity is prevalent. The use of separate model-specific sub-cohorts, necessitated by differing eligibility criteria across the three risk scores, precludes direct head-to-head model comparison; AUC differences across scores should therefore be interpreted with caution. The modest number of observed cardiovascular deaths limits the statistical precision of threshold-specific estimates, particularly the wide confidence interval for the PREVENT Youden AUC (95% CI 0.613–0.793).

The restriction to the cardiovascular mortality, appropriate for model validation using the ICD-10 coded death record in NCHS, excludes the events (heart failure, myocardial infarction), which may have underestimated the true burden of misclassification.

## 6. Conclusions

The traditional ASCVD, PREVENT, and Framingham risk scores fail to demonstrate adequate discrimination and calibration for cardiovascular mortality prediction in cancer survivors. Our results demonstrate that while statistical optimization via Youden-Index recalibration provides a modest improvement in performance metrics, this adjustment introduces a critical clinical paradox by systematically increasing the misclassification of high-risk individuals into the “low-risk” category. This finding underscores that threshold recalibration is insufficient and cannot overcome the fundamental structural failure of applying general population tools to a cardio-oncology population.

## Figures and Tables

**Table 1:** Basic Characteristics.

Characteristics	Master Cohort (n = 1074)	*p*-Value	PCE/ Framingham’s (n = 634)	*p*-Value	Prevent (n = 429)	*p*-Value
Weighted N	5.01		2.9		2.1	
Age (Mean ± SE)	64.89 ± 0.37	<0.001	64.94 ± 0.61	<0.001	63.51 ± 0.7	<0.001
Sex		0.80		0.94		0.99
Male	48% (n = 541)		51% (n = 348)		52% (n = 240)	
Female	52% (n = 533)		49% (n = 286)		47% (n = 189)	
Race		<0.001		<0.001		<0.001
Mexican	1.9%(n = 75)		1.3% (n = 31)		1.2% (n = 19)	
Other Hispanic	2.0% (n = 66)		1.2% (n = 23)		1.1% (n = 19)	
Non-Hispanic White	85.3% (n = 669)		88.7% (n = 445)		88.6% (n = 290)	
Non-Hispanic Black	7.0% (207)		6.7% (n = 117)		6.9% (n = 89)	
Other/multiracial	3.6% (n = 57)		2.0% (n = 18)		1.9% (n = 12)	
Education		<0.001		0.008		0.012
Less than High School	12.9% (n = 231)		16.6% (n = 153)		13.8% (n = 94)	
High School/GED	23.1% (n = 254)		23.1% (n = 149)		23.5%(n = 105)	
Some College	35.3% (n = 340)		35.6% (n = 199)		35.9% (n = 137)	
College Graduate	28.8% (n = 248)		24.5% (n = 133)		26.6% (n = 93)	
Marital status		0.031		0.0006		0.04
Married	74.1% (n = 656)		71.7% (n = 371)		73.5% (n = 255)	
Widowed/Divorced	12.7% (n = 150)		16.2% (n = 113)		12.2% (n = 55)	
Never married	13.2% (n = 148)		12.3% (n = 76)		14.1% (n = 60)	
Taking Hypertension medication (%)	90.6% (n = 975)	0.002	88.4% (n = 571)	0.007	90.1% (n = 392)	0.002
Hypertension, %	82.3% (n = 884)	<0.001	82.1% (n = 634)	<0.001	83.1% (n = 429)	<0.001
Emphysema, %	5.9% (n = 62)	0.001	7.4% (n = 46)	<0.001	6.2% (n = 28)	0.05
Diabetes, %	25.2% (n = 305)	0.005	22.2% (n = 150)	0.18	19.9% (n = 97)	0.37
History of Smoking, %	58.5% (n = 627)	0.003	51.7% (n = 328)	<0.001	48.9% (n = 210)	<0.001
BMI, kg/M^2^ (Mean ± SE)	29.71 ± 0.18	0.07	30.03 ± 0.33	0.003	29.47 ± 0.27	0.17
SBP, mmHg, (Mean ± SE)	131 ± 0.64	0.68	132 ± 1.02	0.43	130 ± 1.07	0.94
Total Chol, mg/dL, (Mean ± SE)	194 ± 1.61	0.009	194 ± 2.18	0.03	198 ± 2.14	0.20
HDL Chol, mg/dL, (Mean ± SE)	51.85 ± 0.60	0.70	53.6 ± 0.9	0.11	51.45 ± 0.85	0.82
Family-income Ratio (PIR) (Mean ± SE)	3.33 ± 0.06	<0.001	3.24 ± 0.07	<0.001	3.32 ± 0.89	0.001

Abbreviations: BMI = Body mass index; SBP = Systolic blood pressure; Chol = Cholesterol; HDL = high-density lipoprotein; PIR = poverty-income ratio; PCE = Pooled Cohort Equations.

**Table 2:** Performance Metrics of Cardiovascular Risk Tools.

Metrics	ASCVD (*p*-Value: <0.001)	Framingham’s (*p*-Value: <0.001)	Prevent (*p*-Value: <0.001)
Standard threshold (7.5%)			
Area Under Curve	0.56	0.53	0.64
Sensitivity	90.5%	90.9%	72.7%
Specificity	40.8%	32.5%	59.4%
High-risk threshold (20%)			
Area Under Curve (AUC)	0.65	0.63	0.51
Sensitivity	61.9%	71.09%	70.5%
Specificity	74.1%	63.1%	89.9%
Youden-optimized Threshold			
Area Under Curve (AUC), *p* < 0.0001	0.68	0.66	0.70
Sensitivity	86.2%	68.5%	73.1%
specificity	48.4%	64.1%	70.3%

At the 7.5% threshold, all models show high sensitivity but poor specificity, with overall low discrimination. Raising the threshold to 20% improves specificity but reduces sensitivity. Using Youden-based thresholds increases AUC across all models.

## Data Availability

The data presented in this study are available in the National Health and Nutrition Examination Survey (NHANES) at https://wwwn.cdc.gov/nchs/nhanes/default.aspx. Mortality linkage data are available through the NHANES-linked National Death Index (NDI) at https://www.cdc.gov/nchs/linked-data/mortality-files/index.html. These data were derived from the following resources available in the public domain: (1) NHANES 2001–2018 survey cycles (https://wwwn.cdc.gov/nchs/nhanes/default.aspx) and (2) NHANES Linked Mortality Files through 31 December 2019 (https://www.cdc.gov/nchs/linked-data/mortality-files/index.html).
